# Esophageal Atresia With/Without Tracheoesophageal Fistula

**Published:** 2012-01-01

**Authors:** Vivek Gharpure

**Affiliations:** Department of Pediatric surgery, Children’s Surgical Hospital 13, Pushpanagari, Aurangabad, 431001, India

## QUESTIONS

1.What clinical and sonographic features should make one suspect esophageal atresia in a pregnancy?

2.What is the quickest method of diagnosing esophageal atresia, with or without tracheo-esophageal fistula (TEF)?

3.What management principles should be followed in a newborn with esophageal atresia ± TEF till surgery is carried out?

4.What difficulties are encountered in a newborn with esophageal atresia ± TEF and right aortic arch?

5.What are advantages and disadvantages of trans-anastomotic tubes in esophageal atresia ± TEF?

6.What are the management options in anastomotic leak in esophageal atresia ± TEF repair?

7.What immediate postoperative care is recommended in a neonate operated for esophageal atresia ± TEF?

8.What long term follow-up is recommended in a survival of esophageal atresia?

9.What management options are available in a child who develops stricture at anastomotic site following esophageal atresia ± TEF repair?

10.What are different investigations in a child suspected to have H type TEF?

## ANSWERS

1.Polyhydromnios, small gastric bubble, dilated upper esophageal pouch [1].

2.Pass a soft blunt tipped rubber catheter through mouth. If it arrests at 10 cm from gum margin, baby has esophageal atresia. Observe the stomach; if it is scaphoid, baby may have isolated esophageal atresia, if it is distended, baby is likely to have TEF [1].

3.Frequent upper pouch suction, semi-erect position, hydration, antibiotics, stop feeds, vitamin K, and warmth [1].

4.Lower esophageal segment concealed under the descending aorta, difficult to locate, anastomosis touched by pulsating aorta, iatrogenic injury to vital structures due to unfamiliarity with anatomy [1].

5.Transanastomotic tubes allow early feeding, prevent catabolism, and prevents double bites of esophageal wall; but can obstruct esophageal lumen and prevent salivary drainage leading to secretions in throat, compromised anastomosis [2].

6.These are; TPN; Gastrostomy/jejunostomy feeds/Transanastomotic tube feeds; Oral feeds if leak is small; Drainage of leak; Esophagostomy and gastrostomy [1,2].

7.These are; Upper pouch suction; Chest physiotherapy; Humidified oxygen; Analgesics; Care of chest tube/NG tube [2].

8.Monthly weight record for first few months; Esophageal calibration for first year; Growth monitoring every year; Investigate for dysphagia; Evaluate for GER if suspicion [2].

9.Anti-reflux measures such as position, Thick formula feeds; H2 blockers; Dilatation; Dilatation and local bleomycin/steroid; Balloon dilatation; Stricturoplasty; Replacement [1,2].

10.Barium swallow; Esophagoscopy/bronchoscopy; CT/MRI [1,2].

## Footnotes

**Source of Support:** Nil

**Conflict of Interest:** None declared

